# Supplementary material for: Are carbon emissions trading and green financial instruments synergistic? -Comprehensive quantitative research based on content analysis
Source: PLoS One. 2024 Mar 7;19(3):e0298601. doi: 10.1371/journal.pone.0298601 (PMC10919601; doi:10.1371/journal.pone.0298601)
Supplement: S1 Data — (ZIP) [file pone.0298601.s001.zip › Data/地级市Z_F工作报告.zip!/╣π╢1⁄2/╔╪╣╪╩╨/└┤╫╘╣1⁄2╓┌║┼í╛┬φ┐╦╩2╛▌═°í┐_2009.docx]

各位代表：

我代表市人民政府，向大会作政府工作报告，请予审议，并请市政协委员和其他列席人员提出意见。

2008年工作回顾

2008年，是我市经济社会发展经受历史罕见的重大挑战并取得显著成绩的一年。在党中央、国务院和省委、省政府及市委的正确领导下，在市人大及其常委会、市政协的监督支持下，市政府团结和依靠全市人民，高举中国特色社会主义伟大旗帜，深入贯彻落实科学发展观，继续解放思想，狠抓第一要务，推进改革开放，克服重重困难，全市经济社会发展取得新的成就，市十二届人大三次会议确定的主要预期发展目标，除生产总值、外贸进出口增长和居民消费价格涨幅控制等指标没有完成外，其余均完成较好。

一、经济保持平稳较快增长，发展质量进一步提高

据统计，全市实现生产总值546亿元，比上年增长10.6%，高于全省平均增长速度。人均生产总值1.85万元，增长10.3%。

工业保持稳步发展。在市场环境极为艰难的情况下，工业增加值完成235.6亿元、增长10.3%，其中规模以上工业增加值增长10.5%，轻工业增长15.4%，民营工业增长22.6%。高新技术产业产值突破 100亿元，增长20%。41个重点工业项目建成投产。与省经贸委签订协议，共建（韶关）装备基础零部件先进制造业基地。

服务业发展加快。第三产业增加值完成205.6亿元，增长13.1%。全社会消费品零售总额达241.3亿元，增长22.8%，创多年新高。城乡市场两旺。沃尔玛、益华百货等著名零售企业在韶关相继开业。居民消费价格基本平稳，指数涨幅低于全国、全省水平。旅游业迅速发展，全年接待旅游者人数1044万人次，旅游总收入52.6亿元，分别增长22.3%和29.4%。

财政金融运行良好。来源于韶关的财政总收入106.4亿元，增长20.8%。全市地方财政一般预算收入36.4亿元，增长21.1%。年末金融机构本外币存款余额674.4亿元，贷款余额221.3亿元，分别比年初增长15.2%和15.1%。

城乡居民收入明显提高。全市城乡居民人均收入8174元、增长11.8%，其中城镇居民人均可支配收入11989元、增长11%，农民人均纯收入4848元、增长12.6%。

县域经济发展良好。县域生产总值增长11.7%，工业增加值增长14.4%，固定资产投资增长30.5%，一般预算收入增长24.9%，均高于全市平均水平。10个县（市、区）地方财政一般预算收入全部实现超亿元。

二、战胜严重自然灾害，农业农村工作稳步推进

在严重低温雨雪冰冻灾害和洪涝灾害面前，我们坚持以人为本，科学应对，全力以赴抗灾救灾；人民子弟兵和广大干部群众并肩作战，打赢了一场全国关注的春运攻坚战，抗灾救灾取得重大胜利，受灾群众得到及时救助，灾害损失降到最低。灾后复产重建任务基本完成。电力、交通、水利、通讯等受损设施全部修复，林业生态修复有序开展。因灾全倒户已全部搬入新居。

农业农村经济平稳发展，新农村建设全面推进。实现农业增加值77.8亿元，增长5.6%。主导产业、特色产业发展壮大，农业产业化进程加快。主要农产品产量增幅明显，其中粮食增长10.5%、肉类增长8.7%。新增一批无公害农产品、绿色食品和有机农产品。市级农业龙头企业销售收入增长15.4%，带动18.1万农户户均增收3705元。改造中低产田3.63万亩，整治农田1.72万亩，改善和恢复灌溉面积6万亩，除险加固小型水库256宗。改造农村公路860公里，镇通行政村公路全部实现路面硬化，提前一年完成省下达任务。帮助农民建成沼气池3.8万个、改造泥砖房和危房1.4万户，解决近8万农村人口饮水安全问题。农村饮用自来水和卫生厕所普及率分别提高到65%和78%。建成200多项农民健身工程和50项“农家书屋”试点工程。中央广播电视节目在农村实现无线覆盖。

三、务实推进“双转移”，外经贸保持稳步增长

认真贯彻落实去年4月汪洋书记在韶关调研时的重要讲话精神和省委、省政府推进“双转移”的战略部署，制定出台《关于推进承接产业转移和劳动力转移工作的实施意见》及有关配套政策。与东莞市共建的东莞（韶关）产业转移工业园以第一名的成绩成功竞得省第二批产业转移扶持资金。全市落实产业转移项目399个，投资总额88.5亿元，实际到位资金68.8亿元，产业转移项目工业增加值增长49.2%。加快打造区域职业技术教育中心，推进农村劳动力培训转移。完成农村劳动力培训2.35万人，增长26.7%。完成农村富余劳动力转移就业6.6万人。转移到本市以外地区就业的劳动力增长20%。转移到本市城镇就业的农村劳动力增长41.3%。

利用外资和进出口稳步增长。实际吸收外资1.82亿美元，增长21.4%，增速居全省第二。外贸出口总额5.8亿美元，增长9.5%。口岸进出口货物总量410.4万吨，增长13.9%。世界玩具经销商巨头美国孩之宝公司在我市设立分公司。坪石电厂B厂、韶关旭日国际有限公司等外资企业增资扩产。参加2008粤港经贸合作交流会、粤港—欧洲经贸交流会、第五届“山洽会”和广东省第二届“农博会”取得丰硕成果。口岸监管水平和现场通关效率提高。韶关新港码头直通香港货运航线复航。韶关至深圳盐田港集装箱铁海联运专列开通。

四、全面加大投资力度，重点项目建设成效显著

全社会固定资产投资完成283.8亿元、增长30.4%，投资率创改革开放以来新高，有力地拉动经济增长。其中基本建设完成投资增长17.5%，更新改造增长81.8%，房地产开发增长39.7%；韶关电网投入建设和改造资金13.1亿元，增长两倍。全市争取国债资金和其他专项资金近6.5亿元，争取省各项资金22亿元。

重点项目完成投资131.5亿元，增长53.4%。交通、电力、城市、工业等领域在建重点项目大多数超额完成年度计划。武广铁路客运专线韶关段、韶赣高速公路粤境段等重大交通工程建设迅速推进。韶钢节能减排、韶关碧桂园、粤北危险废物处理处置中心等工程建设加快。韶钢水处理中心及烧结烟气脱硫工程建成投产。乐昌峡、湾头两大水利枢纽工程和市第二污水处理厂二期、曲江污水处理厂二期、市区防洪排涝二期工程动工兴建。广乐高速、核电、曲江台湾水泥等重大项目前期工作取得可喜进展。

五、着力打造交通枢纽，建设粤北区域中心城市步伐加快

完成《韶关市城市综合交通规划（2005—2025年）》和一批重要区域控制性详细规划。芙蓉新城征地进展顺利。韶关大道、京珠高速公路韶关互通和国道323线市区过境段改造基本完成。武广铁路客运专线韶关站和站前广场、帽峰大桥开工建设。梅乐公路桥梁和路基工程、省道250线桂头至乳源县城段改造工程完工，坪乳公路、国道323线古市至太平段、省道341线龙仙至连平陂头段路面大修工程基本完成。韶关港建设加快，吞吐量增长49%。

创建国家卫生城市一次性通过专家暗访。创建国家园林城市工作取得阶段性成果，成功创建省园林城市。加强环卫基础设施建设，建立环境卫生管理长效机制，城市环境卫生质量全面提高。花拉寨生活垃圾填埋场建成启用并被评为国家一类场，中厂山原垃圾填埋场封闭绿化。整治集贸市场、城乡结合部、城中村和“五小、六乱”成效明显，市容市貌明显改观。全面推进城市绿化，市区建成区绿化覆盖率达40.3%，绿化率37.3%。完成中山公园、帽峰公园、西桥公园和莲花山公园绿化改造，建成市区四大出口和新区道路绿色长廊。芙蓉山国家矿山公园及其博物馆建成并通过国家验收。

六、加强生态文明建设，环境质量持续提高

出台《关于加强生态文明建设的决定》。支持企业节能改造，加快淘汰落后产能。完成国控重点污染源在线监控网络建设。预计全市万元生产总值能耗从上年的1.91吨标煤下降到 1.83吨标煤，下降4.5%；全年二氧化硫、化学需氧量减排均完成省下达的任务，提前两年实现“十一五”减排目标。南枫碧水花城成为全省唯一、全国六个之一的“节能省地型居住建筑综合技术应用科技示范工程”。市区饮用水源水质达标率100%，空气质量达到国家优良标准，在全国109个重点城市排第18位。

林业生态恢复与建设顺利推进。完成造林作业面积40万亩，营造生物防火林带1274公里。以林分改造为生态修复重点，大力推广优良乡土阔叶树种，提高造林质量。累计建立速生丰产林基地300万亩，竹子经营面积215万亩。全市森林覆盖率、活立木蓄积量保持在较高水平。

七、推进改革创新，发展动力进一步增强

在全市“解放思想深化改革年”活动中，各级政府机构继续解放思想，全面深化改革，着力健全有利于科学发展的体制机制。完成第四轮行政审批项目清理。市政府行政许可事项由111项减少到88项。建立政府采购信息预公示制度和政府投资项目公示制度。制定国有土地使用权招标拍卖管理规定。设立市城市管理综合执法局。率先在全省设立人力资源办公室，实施被征地农民养老保险试行办法。完成始兴县集体林权制度改革试点、市级兽医管理体制改革和全市水管单位体制改革。整合市工业、商贸资产经营公司。市政府与四大国有股份商业银行广东省分行签订授信贷款战略合作协议，金融对地方经济发展的支持能力明显提高。

自主创新工作成效显著。获得省级以上科技进步奖和省级科技产业化重大专项均创历史新高，全市获省级以上科技进步奖8项，其中1项获国家科技进步二等奖。完成市科技企业创业园（孵化器）一期改造工程。组织实施“省部企业科技特派员行动计划”，建立汽车零部件、铝镁轻金属材料两个产学研战略联盟。气象、水文和地震监测现代化建设加快，防灾减灾能力明显增强。年末各类人才总量增长12.5%，人才市场信息网络初步建成。乳源东阳光公司经国家批准设立了我市首个博士后科研工作站。

八、全面发展社会事业，民生改善成效明显

全市文明、和谐、安全发展局面进一步巩固。大力加强现代公民道德教育，精神文明建设取得丰硕成果。我市第三次荣获“广东省双拥模范城”称号。罗东元、李大为当选“改革开放30周年感动广东人物”。广大干部群众和社会各界为抗寒抗洪救灾捐款捐物2083万元。汶川大地震发生后，全市捐款3845万元，共产党员交纳特殊党费1210万元，大力支援抗震救灾。全面落实土地利用年度计划执行责任制，开展违法违规用地查处整治行动、矿产资源开发秩序整顿“回头看”行动。安全生产平稳发展，事故起数明显下降。社会治安形势良好，刑事案件、治安案件减少，交通事故、火灾事故得到有效控制。人民调解和信访工作加强，有效化解社会矛盾纠纷。全市没有发生涉法涉诉进京非正常上访事件，社会和谐安定。国防动员、后备力量建设和兵役工作成效显著，国务委员兼国防部长梁光烈上将来韶检查调研后给予充分肯定和高度评价。

教育卫生事业发展取得新突破。全市义务教育学校危房改造全面完成。城乡免费义务教育全面实施。率先在我省东西北地区普及高中阶段教育，毛入学率达86.5%，提前三年实现省委、省政府提出的目标要求。市一中和市中等职业技术学校新校区投入使用。高考各批次上线率、高分层、次高分层人数比例均处我省东西北地区前列。创建国家示范高中工作取得显著成绩。疾病预防控制和卫生应急处置能力增强，连续五年没有发生重大食物中毒事件和甲、乙类传染病暴发疫情。乡镇卫生院和社区卫生服务中心建设加快，医疗卫生服务网络进一步完善。市直医疗机构5个重点专科和23个特色专科建设加快，医疗服务水平和能力明显提高，服务区域扩大。全市临床用血连续10年来自无偿献血，荣获“广东省无偿献血先进城市”称号。农村药品供应网覆盖率达96.8%，药品监督网覆盖率达100%，新型农村合作医疗参合率达98.8%、居全省前列。人口和计划生育工作圆满完成省下达的各项任务，计划生育率比上年提高3.45个百分点，低生育水平保持稳定。

加强促进就业和社会保障工作，困难群体生活进一步改善。全市城镇新增就业5.2万人，失业人员再就业4.07万人，均超额完成年度计划。城镇居民登记失业率控制在3.2%以内。城镇职工基本养老、基本医疗和失业、工伤、生育保险参保人数及基金征缴均超额完成年度任务。近万名早期离开国有企业的职工理顺了参保关系，8.6万多名企业离退休人员再次大幅度提高养老待遇。57万城镇居民参加了基本医疗保险。低保标准和五保供养标准提高，实现了应保尽保。医疗救助、扶贫助学、法律援助等力度不断加大，社会救助体系进一步健全。城镇低收入家庭住房解困工作成效显著，入住廉租房做到当年申报、当年解决。老年人优待范围扩大，残疾人事业、优抚安置和妇女儿童工作进一步加强。

“文化惠民·和谐城乡”工程深入实施。粤北风情歌舞《月姐歌》获首届中国客家文化节金奖，20项文艺作品获国家或省级奖励。民俗瑶族盘王节传承人盘良安成为国家级非物质文化遗产项目代表性传承人，7人成为省级非物质文化遗产项目代表性传承人。《韶关市志》（1988—2000）完成总纂工作。我市运动员参加省青少年锦标赛、省第九届中学生运动会、香港国际武术节和代表省参加全国中老年人太极拳（剑）比赛均取得好成绩，马丽芸作为亚洲唯一的小轮车竞速选手代表中国参加北京奥运会比赛。党的民族政策进一步贯彻落实。引导宗教界服务社会取得新成绩。侨务、外事、对台、档案、统计、审计、人民防空、新闻出版等各项工作有新进步。

九、坚持依法行政，政府自身建设进一步加强

执行重大事项向人大常委会报告制度，认真接受人大及其常委会的法律监督和工作监督，主动接受政协的民主监督，虚心接受群众监督和舆论监督。办理人大代表建议76件、政协建议案3件和委员提案115件，办复率100%，人大代表、政协委员普遍表示满意或基本满意。加强与各民主党派、工商联、无党派人士和人民团体、社会组织的联系，认真听取和采纳社会各界和基层群众的建议和意见。加强政府规章制度建设，颁发规范性文件11个。完成村（居）委换届选举。政务公开、事务公开、村务公开和厂务公开深入推进。

认真贯彻落实《公务员法》，加强公务员教育培训，队伍素质进一步提高。加强党风廉政建设，加大反腐败力度，深入推进商业贿赂专项治理，坚决纠正不正之风，严肃查处腐败案件。加大行政首长问责力度，确保政令畅通。建立覆盖市级、县级和市直机关的效能监察网络。建成全市统一的电子政务基础平台和应用系统，27个部门的160项行政审批事项实现网上申报和审批。市、县两级都有了行政服务中心，并全面推行“一站式”和联办代办、预约服务。健全政府法制和应急机构，完善突发公共事件总体应急预案体系，保障公共安全能力增强。

去年市政府承诺为民办好的八件实事中，全面实行城乡免费义务教育、提高城乡最低生活保障标准和五保供养标准、改造东河职工新村等危旧公房、免费开放韶关国家森林公园、建设市中等职业技术学校新校区已全面完成，兴建五栋廉租房、市职业病防治中心和五里亭消防站均已完成主体工程建设。

各位代表：过去的一年，我市夺取了抗灾救灾和经济社会发展的新胜利，各项建设取得新成绩。国家环保部批准我市为全国首批6个生态文明建设试点地区之一，车八岭国家级自然保护区晋级为世界人与生物圈保护区；与澳大利亚宝活市缔结友好城市，荣获“中国金融生态城市”称号，被列入广东省国民旅游休闲计划试点城市；成功举办张九龄诞辰1330周年系列纪念活动、打造粤北区域中心城市高层论坛、生态发展区落实科学发展观暨“韶关实践”高层研讨会；丹霞地貌申报世界自然遗产工作加快推进；翁源县荣获“中国兰花之乡”称号，乳源、仁化成为广东旅游强县。这些来之不易的成绩和荣誉，大大提高了韶关的城市地位和国际国内知名度。这是在党中央、国务院和省委、省政府的坚强领导下，在中央和省各部门以及各兄弟市的大力支持下，全市人民艰苦努力、团结奋斗的结果。在此，我代表市人民政府，向全市广大工人、农民、知识分子、干部职工，向驻韶人民解放军、武警、消防部队官兵、人民警察和各民主党派、人民团体、社会各界人士，表示崇高的敬意！向长期关心支持韶关发展的港澳台同胞、海外侨胞、驻韶中省单位干部职工和国内外朋友，表示衷心的感谢！

我们也清醒地认识到，我市经济总量偏小、发展提速不快、县域经济落后等长期以来存在的问题仍然突出。受国际金融危机等因素影响，又面临一系列新的困难和问题。主要有：一是部分支柱企业增速减缓、效益下降，导致工业增速回落，全市经济呈下行趋势；二是招商引资力度不大，承接产业转移尚处于初步阶段，发展后劲亟待增强；三是农产品价格呈回落趋势，农业生产成本上升，农业增产、农民增收面临困难；四是部分群众生活困难，老少边穷山区生产生活条件仍然较差，改善民生任务艰巨；五是廉政建设有待加强，个别部门主要负责人因严重腐败被查处，少数部门、单位工作不够扎实，办事能力、服务水平亟待提高。对此，我们必须高度重视，采取有力措施认真加以解决。

2009年工作安排

2009年是新中国成立60周年，是我市推动经济社会发展转入科学发展轨道，推进粤北区域中心城市建设，抵御国际金融危机冲击，实现“十一五”规划目标的关键之年。综观当前形势，国际金融危机对实体经济的冲击影响仍在继续，世界经济衰退已成定局，国内经济下行压力加大，2009年将是我市经济工作遇到挑战最大的一年。我市钢铁、有色金属、电力等支柱产业增产增效面临很大困难，财政减收增支因素明显增多，实现经济平稳较快发展十分艰巨。但是，也要充分认识到，我市和全国全省一样，仍处于发展的重要战略机遇期，经济发展的基本面和长期向好的趋势没有发生改变，仍然面临重大发展机遇和许多有利条件。一是国家推出了进一步扩大内需、促进经济增长的一揽子计划，实施积极的财政政策和适度宽松的货币政策，将形成新一轮的投资热潮。二是我省贯彻落实《珠江三角洲地区改革发展规划纲要》，加快“新十项工程”建设，在今年全社会固定资产投资中，安排与我市直接相关的省重点项目达11项。三是我市加快实施城乡基础设施和产业建设、促进经济持续较快发展的系列措施初见成效，推进“双转移”速度加快。经过改革开放30年的发展，韶关交通区位优势更加突显，城乡基础设施不断加强，推进科学发展有着坚实的物质基础。因此，我们必须始终围绕建设粤北区域中心城市、争当全省山区科学发展排头兵的目标不动摇，坚持以人为本，凝聚广大干部群众的智慧和力量，充分估计各种困难和挑战，不断完善应对措施，通过扩内需保增长、抓项目促发展、调结构上水平、强改革增活力、重民生保稳定，最大限度地化“危”为“机”，促进经济社会又好又快发展。

2009年市政府工作的指导思想是：高举中国特色社会主义伟大旗帜，以邓小平理论和“三个代表”重要思想为指导，深入贯彻落实科学发展观，认真贯彻落实中央和省的部署，紧紧围绕市委十届五次、六次全会确定的发展思路和目标，以深入学习实践科学发展观为动力，以“三促进一保持”为着力点，善于抢抓发展机遇，突出抓工业发展、招商引资和重点项目建设，推进“双转移”工作，加快发展方式转变和结构调整，加快县域经济发展，加快经济社会、生态文明和民主政治建设，着力改善民生，促进社会和谐，努力打造全国生态文明建设示范市、广东生态发展主力军、实施“双转移”战略先行区、山区宜居城乡优选地，在建设粤北区域中心城市、争当全省山区科学发展排头兵的征程上迈开新步伐。

2009年经济社会发展主要预期目标是：全市生产总值增长10%以上，人均生产总值增长与全市生产总值增长基本同步，全面完成省下达的节能减排任务，全社会固定资产投资增长25%，社会消费品零售总额增长15%，地方财政一般预算收入增长8%，实际利用外资增长12%，外贸出口增长10%，城镇登记失业率控制在3.8%以内，居民消费价格指数涨幅控制在4%以内，城镇居民人均可支配收入增长8%，农村居民人均纯收入增长7%，人口自然增长率控制在7.5‰以下。

今年要重点抓好以下九项工作：

一、抢抓机遇扩大投资，扎实推进重点项目建设

最大限度争取国家和省项目、资金支持，全面推进重点项目落实和项目建设，不断加强城乡基础设施建设。发挥政府、企业、银行和社会的积极性，服从国家投资导向，做好项目对接和后续工作，积极创造条件，争取国家和省批准一批、提前开工一批、提早建成一批、及时储备一批重大项目。力争固定资产投资突破350亿元。加强重点项目的服务和监督管理，确保重点项目顺利推进。加快韶赣高速公路粤境段和武广铁路客运专线韶关段建设，开工建设广乐高速公路和赣韶铁路，开展博仁、大广、昆汕等高速公路项目的前期工作。推进国省道改造、北江航道整治、运输站场和港口建设。加快韶钢、丹霞冶炼厂等重大技改项目建设，抓紧对韶关电厂2×60万千瓦机组、曲江台湾水泥、新丰越堡水泥、佛山华厦建陶（新丰）产业转移项目等一批生产性重点项目的跟踪落实，争取早日开工建设。加快改造城市电网和完善农村电网。抓好乐昌峡、湾头两大水利枢纽和市区防洪排涝二期工程建设，加快城乡防灾减灾和水利基础设施建设。

坚持多渠道扩大投入。切实加强财税工作。积极运用财政手段和落实各项税收优惠政策支持企业生产经营，加强税收服务，加大税源培植力度。坚持依法治税，强化税收征管，努力确保财政收入稳步增长。调整支出结构，大力压缩一般行政支出，加大对促进经济增长、改善民生、社会建设、生态文明建设等方面的投入，带动、引导社会和民间投资增长。运用财政补贴、融资担保、以奖代补等手段，引导金融机构扩大信贷总量，加大对重点项目、城市建设和企业改革、改组、改造的支持力度，发展消费信贷，助推经济增长。抢抓国家实行适度宽松货币政策的机遇，健全政银企联席会议制度，完善金融信息平台。积极落实市政府与国有股份商业银行签订的970亿元授信贷款战略合作协议。有效执行国家货币政策，鼓励创新金融产品，提升信贷服务效率和质量，加快金融服务现代化。

二、全力以赴实现工业较快增长，落实全民创业各项措施

加强工业运行分析监测和调节，加强生产要素保障和产、运、需衔接。市级强化对30户重点企业的服务，县级强化对年产值2000万元以上企业的服务，帮助其克服当前困难，充分发挥产能，提高效益。加强对旭日玩具二期工程等新增工业项目的跟踪服务，加快形成新的工业增长点。深化国企改革，引进战略投资者，推进国有工业资产经营和兼并重组。建立健全政府领导班子成员和部门联系服务工业企业、工业项目制度，全力服务好工业发展。力争今年实现工业增加值增长10%以上。

加快自主创新和产业升级步伐，发展高新技术产业，建设现代产业体系。加强与中国科学院及高等院校的战略合作。落实自主创新税收优惠政策，推进产业技术进步和高新技术产业化建设。加快特色产业基地公共技术服务平台建设。组织实施重大项目科技攻关。推进信息化建设、产学研深度合作、县域科技能力建设和实施知识产权战略。抓好韶关高科技产业孵化基地、科技企业创业园（孵化器）建设，扶持专利技术产业化。抓好重点项目技改，加快应用高新技术和先进适用技术改造提升传统产业。推进省市共建（韶关）装备基础零部件先进制造业基地项目建设。加快发展具有比较优势的产业和产品。大力培育发展冶金、机械装备、精细化工、玩具等产业集群，加快建设液压油缸、铝箔化工和有色金属材料等特色产业基地。

落实国家、省、市关于扶持中小企业发展、推进全民创业的政策措施。实施“中小企业成长工程”，重点扶持100家高增长、有自主品牌、市场竞争力和自主创新能力强的中小企业转型升级。全力支持全民创业基地建设。全面清理涉企收费，减轻企业负担。完善中小企业信用担保体系，着力缓解中小企业融资难问题。支持中小企业创新管理体制，与大企业配套发展。加强创业培训，改善创业环境，鼓励自主创业。结合我市产业特色和产业发展规划，制定鼓励创业的产业指导目录，细化具体的扶持措施。加快创业园区建设。加强创业服务，形成“政策扶持、创业培训、创业服务”三位一体的工作机制，努力实现中小企业新发展、全民创业上台阶。

三、强力推进“双转移”，实现招商引资新突破

掀起大规模园区基础设施建设高潮。进一步整合优化园区资源，用好用活省产业转移政策和扶持资金，大力推进产业转移工业园建设，打造一流载体。加强与东莞市合作，加快推进产业和劳动力“双转移”。抓紧完善东莞（韶关）产业转移工业园管理体制和机制，充分发挥省5亿元竞争性扶持资金和莞韶双方政府财政资金的放大作用，做好融资工作，加快园区开发和基础设施建设，加快项目入园步伐，争取早日建成全省示范性产业转移工业园。支持县（市、区）加快特色产业转移园建设，以园区发展带动县域经济发展。

掀起大规模招商引资热潮。围绕落实《珠江三角洲地区改革发展规划纲要》，实施《韶关市承接产业转移区域布局指导意见》。加强与珠三角等主要产业转出地政府、商会、协会和港澳台行业组织的交流和合作，大力推进产业对接。加强与省内外大公司、大企业合作，重点对接珠三角地区汽车、机械、电子、玩具、家俱、精细化工等产业。创新招商思路和方式，重点围绕发展主导产业和物流、旅游、农业等优势、特色产业推进产业招商。突出抓好重大项目和龙头企业招商，着力引进国内外战略投资者。围绕办好今年省“三促进一保持”现场会粤北分片会议，加快南雄精细化工产业聚集。跟踪落实已签约项目，推进重大项目早日落户和开工建设。鼓励外资企业增资扩产。进一步完善和落实利用外资及外贸扶持政策，进一步优化出口商品结构和出口主体结构，推进加工贸易转型升级，保持对外贸易稳定增长。完善韶关铁路口岸“内陆港”，推进韶关口岸多功能通关物流中心建设，提升口岸功能，优化通关服务。

加强人力资源培训和配置，促进劳动力转移就业。整合优化职业技术教育资源，加快职业技术教育基地建设，建设职业技术教育大市，支持、鼓励民办职业教育发展。打造技工教育强市。深化技工教育改革，推进省市共建现代技工教育基地。加强以农村劳动力为主的技能培训，推进就业培训、创业培训和技能提升培训。推进创业富民培训工程。加强企业用工指导，积极引导农村富余劳动力转移就业，促进本地劳动力充分就业。完成农民转移就业培训1.5万人，转移农村劳动力4.5万人。加强人才服务平台建设，创新人力资源管理服务机制，积极引进各类急需人才。

四、围绕增加农民收入，推动农业农村加快发展

推进农村体制改革，激发农业农村发展活力。全面贯彻《中共中央关于推进农村改革发展若干重大问题的决定》和今年中央一号文件精神。制定实施方案，落实省促进农村改革发展实施意见，深化农村综合改革，强化农业基础地位。稳定和完善农村基本经营制度，稳定农村土地承包关系。落实最严格的耕地保护制度。健全集体建设用地使用权和农民土地承包经营权流转制度，尊重农民主体地位，培育发展土地流转市场，建立健全土地流转机制。全面推进集体林权制度改革，加快建立现代林业产权制度。

积极发展现代农业，促进农民增收。优化农业产业结构，加快发展以沼气为纽带的生态农业，做强养殖业，做优特色种植业，扩大优质农产品生产规模。抓好百万头生猪工程和一亿只家禽工程。重视抓好粮食生产，确保粮食安全。加快粤北现代农业示范园区和粤港农产品加工物流中心建设。发展种植名优珍贵树种、竹子等特色林业。大力发展农林产品加工业，提高农林产品附加值。加快发展农业龙头企业和农民专业合作组织，推进农业产业化。实施优质农产品认证制度和农产品商标战略，鼓励农产品注册商标、申报知名商标和国家地理标志产品。强化农业技术推广公益性服务，加强动物重大疫病防控体系和气象灾害防御体系建设，健全完善农业社会化服务体系，提高农产品质量安全。加强农田水利建设，推进病险水库除险加固、小流域综合治理、中低产田和灌区改造，按计划完成城乡水利防灾减灾工程建设。加快推进农业机械化和农业信息化。拓展农村劳动力转移就业渠道，发展农村二、三产业，努力增加非农收入。

大力发展农村公共事业，加快推进新农村建设。抓好农村规划、建设和管理，大力推进村庄整治。着力解决农村食水安全问题。抓好11个建制镇、110个自然村创建宜居村镇示范点工作。推进有条件的自然村公路路面硬化，完成400公里农村公路建设。推进农村薄弱学校改造。健全新型农村合作医疗制度。推进乡镇综合文化站建设，继续做好广播电视“村村通”工作，实施省级农村广播电视节目无线覆盖工程，加快“农家书屋”工程和农民健身工程建设。积极探索建立新型农村社会养老保险制度。推进扶贫开发，大力支持水库移民居住区、革命老区、边远山区、民族地区和石灰岩地区加快发展。推进文明村镇、和谐村镇和“平安乡村”创建活动，促进农村社会和谐稳定。

五、发展大旅游产业，推动现代服务业持续快速发展

以广东省推进全国旅游综合改革示范区建设为契机，与省旅游局、省信息产业厅紧密合作，积极争取政策、资金支持，抓紧修编全市旅游发展总体规划，整合旅游资源，着力引进战略投资者对大丹霞、大南华、大南岭等重点景区进行综合开发，加快旅游景区基础设施和服务配套设施建设，加快打造粤北生态旅游休闲度假基地。积极推进“丹霞地貌”申报世界自然遗产以及丹霞山创建国家5A级景区、全国文明景区工作。支持乳源打响“世界过山瑶之乡”旅游品牌。抓住国家和省启动“2009中国生态旅游年”的机遇，认真策划举办“绿色韶关、生态韶关、魅力韶关”为主题的特色节庆活动。做好广东省国民旅游休闲计划试点城市工作。加快省旅游信息化示范区建设，用信息技术提升传统旅游业。推进旅游购物市场、旅游休闲娱乐街区建设，鼓励引导旅游商品开发生产，拓展购物、娱乐、餐饮等消费环节。加强旅游宣传推介和区域合作交流，巩固珠三角地区主客源市场，拓展华中、华北、华东客源市场，开发日韩、东南亚等国际市场。推进红三角区域旅游合作。实现全年接待旅游者人数、旅游总收入分别增长10%和12%。

扩大城乡消费需求。继续优化城乡商业网点，推进实施“千街万巷”、“万村千乡”市场工程，落实“家电下乡”等消费政策。继续整顿规范市场经济秩序，加强对涉及人民群众身体健康和生命安全的食品药品等重点产品的监管，严厉查处制售假冒伪劣产品违法行为，加强市场价格监管，稳妥推进价格和收费改革，营造良好消费环境。落实国家和省的相关政策，鼓励住房消费，引导企业主动应对市场变化，促进房地产业持续健康发展。加快物流基础设施和公共信息平台建设，整合物流资源，扶持促进重点物流企业做大做强。加快粤北保税物流中心、韶关出入境货物速递中心、粤北粮油交易中心建设。加快建设韶关信息服务港，推进电子商务发展，培植金融、会展、保险等现代服务业。加快改造传统服务业，大力发展社区服务业，刺激消费需求，促进灵活就业。

六、加快推进芙蓉新城建设，增强市区聚集功能

强化规划引导和控制，推进城市建设与产业布局、交通旅游、社会发展紧密衔接。做好《韶关公路运输枢纽总体规划》、《市区历史文化名城保护规划》、《市区建筑风貌控制规划》、《浈江区黄竹地区发展规划》等编制工作。突出抓好芙蓉新城建设，全面推进新城征地拆迁工作，加快村民安置区建设。继续抓好武广铁路客运专线韶关站及站前广场、芙蓉新城综合客运枢纽站等基础设施建设。统筹资源布局，积极引导城市新增需求向芙蓉新城转移。

加快城区建设改造，增强城市功能。继续完善城区道路、给排水管网，加快帽峰大桥、北伐纪念馆、张九龄纪念公园、望韶楼等工程建设，推进林桥坑一期整治、武江路河堤加固改造和污水管网工程、马坝河防洪堤二期工程，建设市区防洪堤芙蓉新城段。优先发展城市公交。推进道路交通站场建设，加快建设大坑口港区和白土港区。加强“三江六岸”城市景观和芙蓉山国家矿山公园等城市公园建设，凸显山水名城特色。

继续推进城市创建工作，建立健全长效管理机制。突出抓好城镇环境保洁、集贸市场管理、“五小、六乱”和卫生死角整治，加强病媒生物防治，全面完成创卫整改任务。加强园林景观建设，提高绿化水平，确保人均公共绿地、绿化覆盖率、绿地率等指标全面达到国家园林城市标准，力争创建国家卫生城市和国家园林城市成功。启动创建全国文明城市工作。加强对外合作交流，推进缔结友好城市工作步伐。

七、加强节能减排和环境保护，推进生态文明建设

加强节能降耗，推进资源节约集约利用。做好全国生态文明建设试点市工作。严格执行主要污染物排放总量控制制度，建立污染减排倒逼机制，确保完成年度主要污染物削减任务。推行清洁生产，推进循环经济试点工程，扶持培育一批清洁生产企业。严格执行节能问责制和“一票否决制”，实施《韶关市节能中长期专项规划》，鼓励和引导企业实施节能降耗，开展全民节能行动。安排专项资金，重点支持节能重点领域、重要产业的节能技术研发、推广和利用。抓好火电、水泥等工业“上大压小”项目建设，加快淘汰落后产能，鼓励发展高效、节约、循环的产业和产品。大力推广使用农村户用沼气和太阳能热水器。建立建筑节能技术标准体系，抓好节能示范项目建设。认真实施《广东省建设节约集约用地试点示范省工作方案》，落实政府保护耕地第一责任及部门共同监管制度，切实抓好土地开发整理、“三旧”改造、闲置地处置，推进节约集约用地。加强水、土地、矿产等自然资源管理，禁止无序开发。

加强生态建设，推进森林生态市创建。继续开展林业生态修复，大力开展植树造林和封山育林，完成年度荒山造林任务。加强重要生态功能保护区和自然保护区建设。加强石漠化综合治理、矿区生态恢复、重点流域水生态保护和地质灾害防治。抓好森林资源综合管护，保护生物多样性，推进全国环境优美乡镇、全国绿色小康县（村、户）等创建活动，创建宜居村镇。

加强环境基础设施建设，推进生态环境综合治理。重点抓好市第二污水处理厂二期、曲江污水处理厂二期和翁源、乳源、仁化、乐昌坪石污水处理厂建设。加快火力发电厂脱硫工程建设。加快固体废弃物处置产业的发展，加强各类固体废物污染的防治。加强建设项目环保监管工作。抓好水污染和土壤污染的综合防治。建立完善污染事故应急处置系统。建立健全符合生态文明建设要求的指标体系和考核办法。

八、着力保障和改善民生，全面发展社会事业

实施更加积极的就业政策，落实就业援助措施，完善四级公共就业服务体系建设，提升公共就业服务能力。组织实施各类培训工程，以培训促进就业，以创业带动就业。支持劳动密集型企业发展。大力开发就业岗位。着力帮助失地农民和返乡农民工就业、创业。实现全年城镇新增就业4.5万人，失业人员再就业3.5万人。扩大被征地农民养老保险覆盖面，大力推进社会保险扩面征缴，实现城镇未成年人、低保户和残疾人基本医疗保险应保尽保。进一步完善医疗、工伤和失业保险制度，推进城镇居民基本医疗保险。做好基本养老保险关系省内转移接续工作。建立健全以城乡低保为基本内容的社会救助制度。拓展和规范法律服务，扩大法律援助覆盖面。继续开展“智力扶贫”，安排农村困难家庭子女免费入读中等职业和技工学校。完成全市敬老院改造。完善住房保障制度，大力发展廉租房和经济适用住房，建立健全城镇住房解困工作目标责任制。完成沙洲尾1.4万平方米廉租住房和东岗岭1.8万平方米经济适用住房主体工程建设。大力开展扶贫济困活动，推进社会福利和慈善事业发展。突出办好十件实事：1、完成农村劳动力转移就业培训1.5万人。2、完成19万农村人口食水安全工程。3、建设市人力资源市场。4、完成村庄整治110个。5、建设帽峰大桥。6、帮助市区水上居民上岸定居。7、建设市儿童福利大楼。8、建设全市旅游公共标识系统。9、市区更新、增添一批公交车辆。10、异地重建韶关市中心血站。

扎实开展深入学习实践科学发展观活动，形成全市上下推动科学发展的强大合力。积极推进社会主义荣辱观和现代公民道德教育，加大科普力度，深入开展群众性精神文明创建活动，积极倡导科学、健康、文明的生活方式，提高城乡文明素质。结合开展庆祝建国60周年活动，加强革命传统、爱国主义和社会主义教育。广泛开展各类社会公益活动，发展志愿者事业，努力培育知荣辱、讲正气、守法纪、重诚信、促和谐的社会主义文明新风尚。

坚持优先发展教育，启动创建教育强市工作。调整优化中小学校布局，推进义务教育规范化学校建设，促进城乡义务教育均衡发展。深化教育体制改革，进一步改善办学条件，加强教师队伍建设，实施素质教育，巩固提高义务教育发展水平和普及高中阶段教育成果。加快发展幼儿教育，提高镇村幼儿入园率。支持韶关学院等高校发展，提升高等教育质量。推进各类教育均衡发展。深化医药卫生体制改革，加强医疗卫生服务体系建设，增强疾病预防控制、卫生应急和医疗救治能力。合理配置卫生资源，进一步完善农村三级卫生服务网络，加快城市社区卫生服务中心建设。坚持专科强院发展道路，推进医疗服务区域中心建设。深化文化体制改革，推进文化事业发展，加快构建公共文化服务体系。抓好文化惠民工程，完善文化站配套设施。完成市直艺术院团改革。加强文物和非物质文化遗产保护。承办好2009广东禅宗六祖文化节。推进数字电视整体平移工作。积极开展群众文体活动，推进群众体育、竞技体育和体育产业协调发展。办好第十三届市运会。积极做好承办省第四届少数民族传统体育运动会筹备工作。加大创建“两无”工作力度，进一步综合治理出生人口性别比偏高问题，继续稳定低生育水平。重视发展老龄事业，关心支持残疾人事业。推进我市妇女儿童发展纲要规划实施，切实保障妇女和未成年人合法权益。不断完善国防动员体系，加强民兵预备役工作，深入开展双拥共建活动，打造“双拥”工作新亮点。促进少数民族地区经济社会又好又快发展。依法依规管理宗教事务。进一步做好统计、审计、外事、侨务、对台、史志、档案、气象、水文、地震监测、人民防空、红十字会和无偿献血等工作。

九、加强民主法制工作，建设廉洁高效的服务型政府

认真落实人大及其常委会的各项决议决定，主动向人大及其常委会报告工作，接受监督。加强与人民政协的联系并向其通报工作，支持人民政协和政协委员履行职责。提高人大议案、代表建议和政协建议案、委员提案办理质量。广泛听取社会各界和人民群众的意见和建议，认真接受群众监督和舆论监督。充分发挥各人民团体的桥梁纽带作用。深入贯彻《全面推进依法行政实施纲要》和《行政许可法》，进一步完善重大决策程序。推进政务公开和政府信息公开，提高政府运作的透明度，保障公民对公共事务管理的知情权、参与权和监督权，促进依法、科学、民主决策。

扎实推进惩治和预防腐败体系建设，落实中央《建立健全惩治和预防腐败体系2008—2012年工作规划》，从源头上遏制腐败。进一步完善对权力运行的监督机制，重点加强对领导干部、人财物管理使用的监督和制约。深入开展执法监察、廉政监察和效能监察。深入开展纠风工作，大力治理商业贿赂，全面清理“小金库”。健全经济责任审计制度，扩大审计结果公开范围。坚决查处各类违法违纪案件，依法严惩腐败分子。加强对公务员的培训、管理和监督，完善考核体系和奖惩机制，努力建设一支政治坚定、业务精通、勤政廉洁、作风优良的公务员队伍。

完善机关绩效评价机制，加大公众评议力度，着力改进机关作风，提高办事效率和服务水平。建立完善重大决策督办制度，加大效能投诉查处力度，确保政令畅通，维护企业、群众的合法权益。按照中央和省的统一部署，适时实施新一轮地方政府机构改革，优化行政资源配置。稳妥推进事业单位分类改革。加强行政服务中心建设。推进政府上网工程，扩大网上服务领域。加快行政审批电子监察系统建设，推进省、市、县三级联网监察。

加强应急物资储备和应急平台建设，完善应急预案体系，全面提高应对和处置突发公共事件的能力。加强基层政权和社区建设，抓好“六好”平安和谐社区和村务公开民主管理示范单位创建。强化安全生产目标管理和专项治理，进一步减少事故发生。加强信访维稳、行政复议和法制宣传教育工作，健全人民调解、行政调解和司法调解联动机制，及时排查和化解各类不稳定因素。严厉打击严重刑事犯罪，加强社会治安综合治理，推进和谐韶关、平安韶关建设，维护社会稳定。

各位代表！我们走过了改革开放30年的光辉历程，开始新的征途。展望未来，我们充满必胜信心。让我们更加紧密团结在以胡锦涛同志为总书记的党中央周围，以前所未有的工作责任心和紧迫感，脚踏实地，奋勇争先，创造优异的成绩向新中国成立60周年献礼！
